# MUC1 and MUC5AC Acting on* Helicobacter pylori*-Related Deficiency and Solid Syndrome of Spleen and Stomach

**DOI:** 10.1155/2018/9761919

**Published:** 2018-04-23

**Authors:** Ling Hu, Wanqun Chen, Ming Cheng, Ting Zhang, Shaoyang Lan, Peiwu Li, Weijing Chen

**Affiliations:** ^1^Institute of Gastroenterology, Guangzhou University of Chinese Medicine, Guangzhou 510405, China; ^2^Chongqing Hospital of Traditional Chinese Medicine, Chongqing 400037, China; ^3^The First Affiliated Hospital, Guangzhou University of Chinese Medicine, Guangzhou 510405, China

## Abstract

To investigate the relationship of MUC1, MUC5AC, and the syndrome of spleen and stomach, 109 subjects (34 peptic ulcer (PU), 62 chronic gastritis (CG), and 13 healthy volunteers (CON)) were included. All the subjects included were surveyed with questionnaire to classify them into damp-heat syndrome of spleen and stomach (DHSS), spleen-qi deficiency syndrome (SQD), and CON, examined by gastric endoscope, and biopsied. Rapid urease and methylene blue staining (MBS) were performed on every subject to diagnose for* Helicobacter pylori* (Hp) infection, and both were defined as Hp-positive. Hematoxylin and eosin (HE) staining was performed on every specimen to explore the histomorphology, inflammatory degree, and inflammatory activity of different groups; then Elivision™ plus kit was used to test the expression of MUC1 and MUC5AC. All the results of digital images were reviewed by two experts blindly. The inflammatory degree with Hp infection was higher than those uninfected or CON, but no significant difference was found between DHSS and SQD. And the expressions of MUC5AC with positive Hp was higher than those with negative Hp or CON regardless of the deficiency and solid syndrome of spleen-stomach but not for MUC1. We speculate that the deficiency and solid syndrome of spleen-stomach is a condition like Tai Ji symbol of dynamic equilibrium, showing the higher expression of MUC5AC but no change of MUC1 in the circumstance of Hp infection.

## 1. Introduction

It is well established that* Helicobacter pylori* (Hp) is the main etiologic factor in a range of pathologies including chronic gastritis (CG), peptic ulcer (PU), and even gastric cancer (GC) [1, 2]. The theory that intestine GC is a multistep process starting with CG and progressing through atrophy, intestinal metaplasia (IM), and dysplasia triggered by Hp is well known [3]. Therefore, the multiple and complicated disruptions of organism caused by Hp have long been the research highlights.

Previous studies have shown that infection with Hp is able to induce a cascade of innate and adaptive immune response for the gastric mucosa [4, 5]; furthermore, triggered by Hp infection, the alteration of mucins (MUCs) in the gastric epithelium and their functions have been widely investigated [6–9]. In particular, MUC1 and MUC5AC are believed to be the most critical proteins for protection from Hp or in the process of carcinogenesis, which have previously been assumed. On one hand, based on the in vitro and mouse model research, studies have elucidated that Hp dwelling exerts the reduction of MUC1 expression due to the mucosal barrier injury [9–11], whereas, on the other hand, it was suggested that glycan-rich niche produced by mucins provides a preferential binding point for Hp [9]; notably, the abnormal expression of MUC1 was recognized as oncogene in the development of gastric carcinomas [12, 13]. In contrast, the expression of MUC5AC was proven to be reduced in the gastric endoscopic biopsy specimens with Hp infection [14], and the significant decrease was demonstrated to represent a marker of worse survival probability in GC [15]. In conclusion, as the critical mucin and the major receptor for Hp, the dual role of MUC1 and MUC5AC can be considered as powerful two-edged sword.

On the basis of holism concept and syndrome differentiation, Chinese medicine (CM) has been paid more attention recently [16, 17]. And syndrome or Zheng differentiation is the critical step in clinic; thus our team has long been contributing to the research of solid and deficiency syndrome of spleen and stomach. The establishment of the animal model of damp-heat syndrome of spleen and stomach (DHSS) and the diagnostic standards of spleen-qi deficiency syndrome (SQD) laid a solid foundation for the research on the relationship of inflammatory cytokines and syndromes triggered by Hp [18–21]. Therefore, based on the previous studies, we hypothesize that, in the circumstance of Hp infection, MUC1 and MUC5AC may be involved in solid or deficiency syndrome of spleen and stomach.

## 2. Materials and Methods

The present study was approved by the ethics committee of the First Affiliated Hospital of Guangzhou University of Chinese Medicine, and each individual gave signed informed consent.

### 2.1. Materials and Chemical Regents

The gastric endoscope was obtained from Olympus (Nagano, Japan), and the rapid urease was obtained from Kedi (Guangzhou, China). Monoclonal antibody of MUC1 and MUC5AC was purchased from ZSGB-BIO (Beijing, China), and the cytokine assay of Elivision plus kit was obtained from Maxin (Fujian, China). The microscope used in this study was Olympus (Nagano, Japan).

### 2.2. Subjects Selection

From March 2010 to March 2011, 109 pairs of gastric endoscopic biopsy specimens, including 34 PU, 62 CG, and 13 healthy volunteers, were collected from the First Affiliated Hospital of Guangzhou University of Chinese Medicine. The diagnostic of CG and PU was reference to the consensus of CG in China and diagnostic criteria of Lancet, respectively [22, 23]. The diagnostic standards of Hp infection were followed by the associated detection technique [24–26]. Moreover, by reference to the previous study of our team and the state administration of CM in 2002, the diagnostics of DHSS and SQD were established [21, 27]. Similarly, the inclusion and exclusion criteria were the same as our previous research [21, 28]. And the detailed information of all subjects included in this study is described in Table 1.

In order to evaluate the symptoms and sighs, all the subjects included in the present research were surveyed by two experts of our team in a scientific, objective, and professional way.

### 2.3. Sample Preparation

All the subjects included were asked to be examined by the gastric endoscopy, and two samples (a pair) were collected from each stomach antrum, namely, from the greater curvature and the opposite position, respectively. In order to make the initial diagnosis of Hp infection, one of the samples was tested with a rapid urease immediately; simultaneously, the other was fixed with formalin. Aiming to test Hp infection in a histological way, the specimens were paraffin-embedded and sectioned, and methylene blue staining (MBS) was performed. Only those with double positive results of a rapid urease and MBS were defined as Hp positive.

In order to observe the morphological characteristic of each specimen, hematoxylin and eosin (HE) staining was prepared routinely. In accordance with the consensus of 2006 [23, 29], two professional experts examine the degree of inflammation, inflammatory activity, and Hp infection condition as none, mild, moderate, and severe independently and blindly.

### 2.4. Mucins Protein Assay

Following the Elivision plus kit manufacturer's instruction, the expression of MUC1 and MUC5AC was detected by immunohistochemistry (IHC). As the same method as the HE staining observation, additionally referring to the diagnostic standards of IHC [30, 31], the expression of mucins protein was evaluated.

### 2.5. Statistical Analysis

By using SPSS software version 22.0 for Windows, a two-tailed *P* < 0.05 was defined as statistical significance in this study. And related data was expressed as the mean ± standard deviation; one-way ANOVA or *t*-test was applied. For the clinical parameters, Pearson's *χ*^2^ test was conducted, and with quantitative variables, Chi-square test with 95% confidence intervals was screened.

## 3. Results

### 3.1. The Clinical Parameters of the Subjects Included

As shown in Table 1, no statistical significance of different groups exists among gender, diseases, or age, which is the reasonable prerequisite of the following experiment.

### 3.2. Hp Infection of Gastric Mucosa with MBS

**Figure 1:**
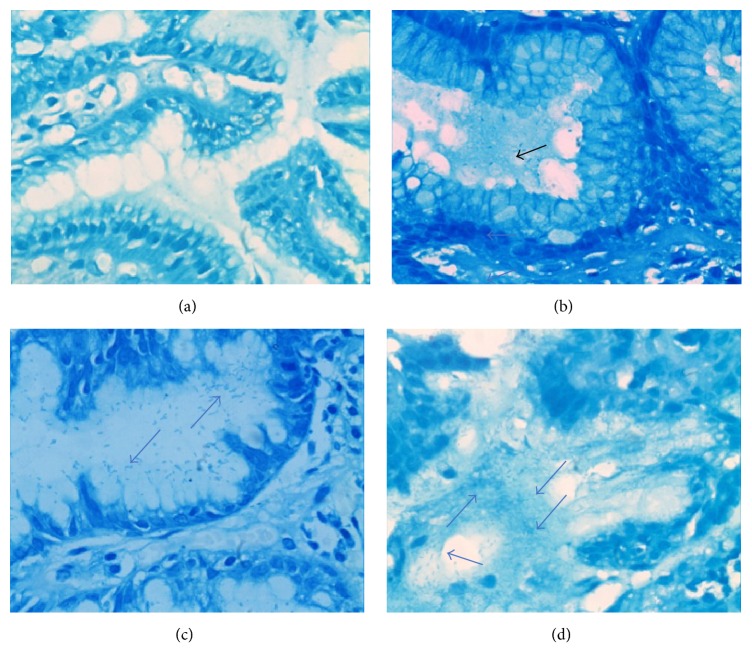
Methylene blue staining (MBS) of gastric mucosa (×400): (a) no* Helicobacter pylori* (Hp) infection; (b) mild infection: spiral-shaped or small blue rods (arrows) Hp are visible in the gastric pit or mucus layer (arrow); (c) moderate infection; (d) severe infection. The arrow refers to Hp infection in the gastric mucosa.

Aiming to increase the sensitivity and specificity for Hp infection test, the specimen was detected with rapid urease; additionally, MBS was performed routinely, and both were defined as positive Hp infection. As shown in Figure 1, under microscope, Hp is shown like curved or spiral bacillus in the epithelial surface, mucus layer, or gastric pits. According to the different amount of Hp dwelling, none, mild, moderate, and severe infections were screened.

### 3.3. Inflammatory Condition of Gastric Mucosa in Different Groups

**Figure 2:**
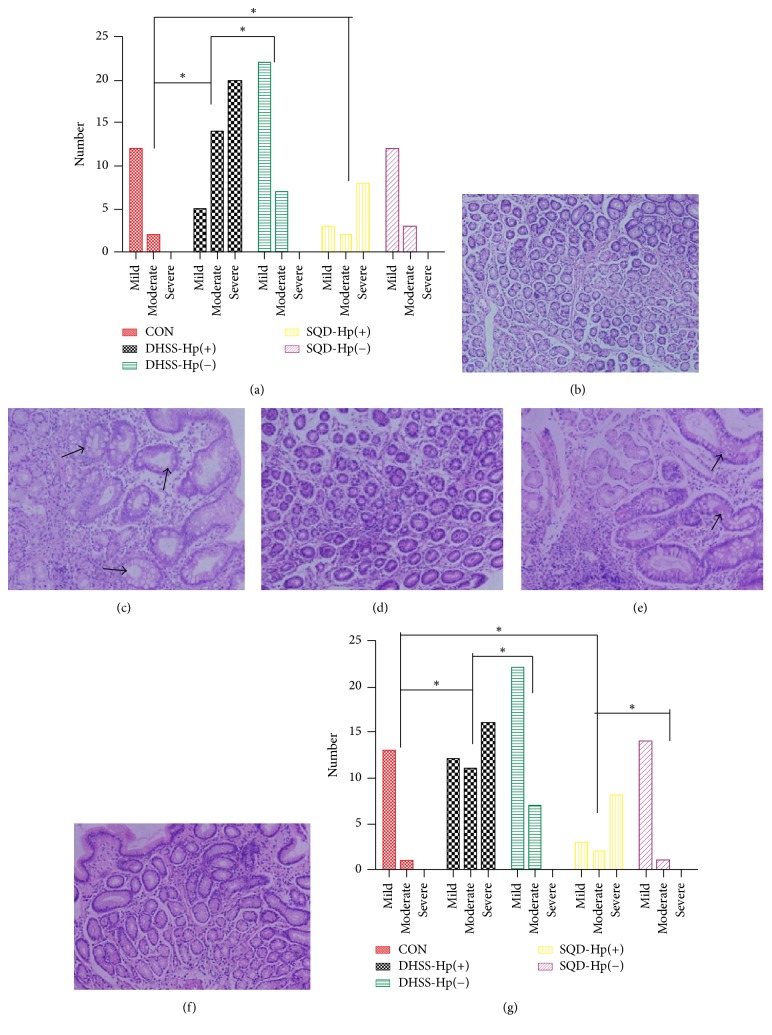
Histomorphology and inflammatory condition of gastric mucosa samples: (a) inflammatory degree of different groups, ^*∗*^*P* < 0.05; (b) normal gastric mucosa of control group (CON): inflammatory cells are rare and gastric glands arrange in order (×100); (c) histomorphology of damp-heat syndrome of spleen and stomach (DHSS) with Hp infection: a few number of inflammatory cells infiltrate the gastric mucosa, with intestinal metaplasia visible (IM) (arrow) (×100); (d) histomorphology of DHSS without Hp infection: several inflammatory cells and IM or dysplasia present infrequently (×100); (e) histomorphology of spleen-qi deficiency syndrome (SQD) with Hp infection (×100); (f) histomorphology of SQD without Hp infection (×100); (g) the inflammatory activity of different groups, ^*∗*^*P* < 0.05. The arrow refers to Hp infection in the gastric mucosa.

With HE staining of specimens, histomorphology appearance of different groups is shown in Figures 2(b)–2(f). In conclusion, compared with CON and those with negative Hp, the subjects with Hp infection had more inflammatory cells infiltrating (*P* < 0.05), even with intestinal metaplasia (IM) or dysplasia, regardless of DHSS or SQD. However, no statistical difference was found between DHSS and SQD. By reference to the criteria of IHC [30], the inflammatory degree and activity were present in Figures 2(a) and 2(g).

### 3.4. Expression of MUC1 and MUC5AC

**Figure 3:**
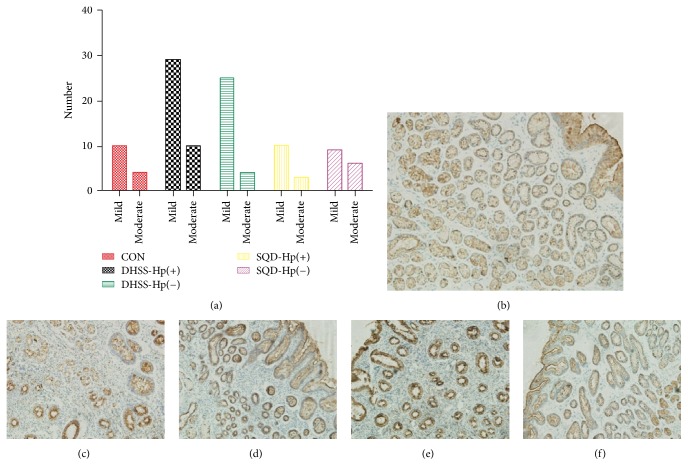
Expression of MUC1: (a) expression of MUC1 in different groups: no statistical difference was observed; (b) expression of MUC1 in control group (CON) (×100); (c) expression of MUC1 in damp-heat syndrome of spleen and stomach (DHSS) with Hp infection (×100); (d) expression of MUC1 in DHSS with Hp negative (×100); (e) expression of MUC1 in spleen-qi deficiency syndrome (SQD) with Hp infection (×100); (f) expression of MUC1 in SQD with Hp negative (×100).

**Figure 4:**
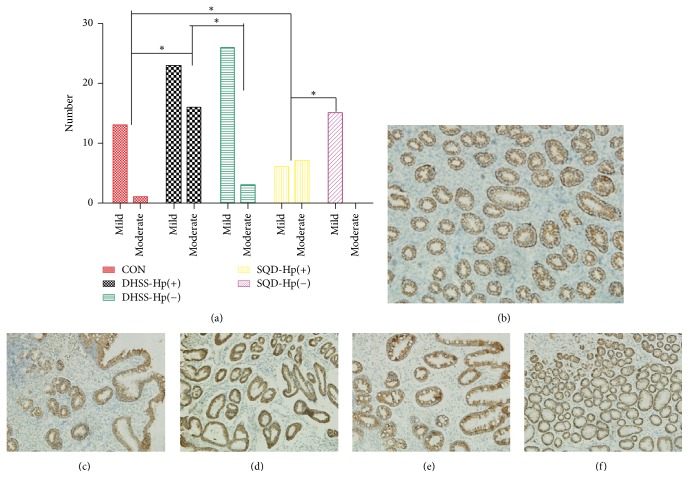
Expression of MUC5AC: (a) expression of MUC5AC in different groups, ^*∗*^*P* < 0.05; (b) expression of MUC5AC in control group (CON) (×100); (c) expression of MUC5AC in damp-heat syndrome of spleen and stomach (DHSS) with Hp infection (×100); (d) expression of MUC5AC in DHSS with Hp negative (×100); (e) expression of MUC5AC in spleen-qi deficiency syndrome (SQD) with Hp infection (×100); (f) expression of MUC5AC in SQD with Hp negative (×100).

As shown in Figures 3 and 4, marked by Elivision plus kit, positive expression of MUC1 and MUC5AC was stained with yellow-brown color. And MUC1 was predominantly screened in gastric epithelium mucosa and gland cells, while MUC5AC was mainly detected in the crypt of gastric epithelial gland cells. There was statistical significance for the expression of MUC5AC between the group of DHSS with Hp infection and CON and also between the group of SQD Hp positive and CON (*P* < 0.05). In contrast, no significance existed for the expression of MUC1.

## 4. Discussion

Nowadays, based on the acknowledgement and communication between CM and Western medicine, some theory has been suggested. For example, Western medicine has been paid more attention to recognize the individual spiritual fulfillment and proposed diagnostic strategies as system-based diagnosis [17, 32]. On the other hand, for exploration of the spirit of CM, microcosmic point of view of CM has been investigated [33, 34]. Our team has long been contributing to the research on the essence of deficiency and solid syndrome of spleen and stomach; previous studies on the elucidation of the theory of DHSS and SQD have laid solid foundation for this research [28, 35].

As we all know, Hp has been considered as I carcinogen for human beings [36], and continuous infection can cause CG, PU, IM, dysplasia, and even gastric malignancy [3]. In the point view of CM, Hp invasion and colonization in stomach are the representative of evil-qi, while the protection barrier for Hp (gastric epithelium) is the delegate of healthy-qi, whereas mucins (MUCs) are the critical polyprotein components of the gastric epithelium. Consequently, we investigated the relationship of MUCs and the deficiency and solid syndrome of spleen and stomach triggered by Hp infection in order to imply the syndrome spirit of CM.

The human MUC consists of secreted mucins and transmembrane ones, in which MUC1 is transmembrane glycoprotein involved in the signal of epithelial-mesenchymal transition (EMT), while MUC5AC is secreted protein [13], recognized as the major receptor for Hp in the human stomach [37]. There is evidence that MUC1 is a physical barrier to protect gastric mucosa from Hp dwelling in murine infection model and in vitro experiment; in turn, by injuring the physical barrier, Hp infection would decrease the expression of MUC1 [9–11]. However, for human gastric tissues, studies have shown that no significant association exists between the expression of MUC1 and Hp infection, which is in accordance with our results in this study, whereas it was overexpressed in the dysplasia and adenocarcinoma tissue, showing its carcinogen characteristics [12, 38].

The deficiency and solid syndrome of spleen and stomach is the dynamic procession of transportation and transformation for spleen with the whole body. And the deficiency and solid syndrome is just another form of Yin and Yang like Tai Ji symbol, both of which grasp each other but are also present within each other. Namely, the two sides alter as ecologic succession to sustain dynamic equilibrium, and change of some critical element may disturb the homeostasis. Although, compared with CON, the expression of MUC1 was not different, we still cannot conclude that MUC1 has no association with SQD and DHSS, because Hp inhabitant in gastric epithelium is like wind invading the skin barrier; of course, there is no change for the skin in a modern point of view, but it is considered to be due to insecurity of the interstices in CM.

In addition, previous study has shown that only small portion of subjects infected with Hp will develop into malignancy, and the abnormal expression of MUC1 acts like oncogene [12, 39]. Therefore, we cannot deny that MUC1 sustaining may be the related element of dynamic equilibrium regardless of the deficiency and solid syndrome.

On the other hand, for the expression of MUC5AC, results show some difference and even contradiction in different teams. By detection of the expression of endoscopic biopsy with IHC, Kocer et al. [14], have demonstrated that MUC5AC was decreased in patients with Hp positive, with its localization in the superficial epithelium, upper parts of gastric glands, and dysplastic areas but not in IM. In contrast, with the same method, Park et al. [40], elucidated that MUC5AC was overexpressed in IM of young adults compared to normal and Hp-infected gastric mucosa of children. In consistence with our study, as Figure 4 shows, the extent of MUC5AC expression with Hp infection was more than that with no infection. We speculated that it might result from the fact that the specimens with Hp positive included in our study showed more IM and dysplasia compared with CON and those with no Hp infection (Figure 2).

In the circumstance of Hp long-time inhabitant in gastric epithelium, to protect the gastric epithelium from deeper damage, MUCs (mainly MUC5AC and MUC1) serve as healthy-qi resistant to Hp. From the holism perspective, the individual syndrome can demonstrate the two sides: deficiency (SQD) and solid (DHSS), but it is another relative homeostasis for gastric epithelium to get dynamic equilibrium showing higher expression of MUC5AC and no change of MUC1. We speculate that it may be the evidence that stomach-qi was still strong even though subjects showed SQD until the equilibrium was broken when showing abnormal expression of MUC1. Consequently, the deficiency and solid syndrome is just relative condition like Tai Ji symbol.

However, with Hp positive, no statistical significance was found between the groups of DHSS and SQD (*P* > 0.05); we still cannot conclude that MUC5AC gets no correlation with the difference of deficiency and solid syndrome of spleen and stomach because of the limited subjects. Thus, in the following, the extension of included samples is the critical approach. Furthermore, investigation of the single nucleotide polymorphisms (SNPs) for the genotype of MUC1 and MUC5AC may be another method to imply the essence of deficiency and solid syndrome of spleen and stomach.

## Figures and Tables

**Table 1:** Clinical parameters for all included individuals.

Group	Number	Gender		Diseases		Age (Y)
		M	FEM	PU	CG	$\overset{-}{\chi }\pm S$
DHSS	68	40	28	32	36	39.84 ± 10.44
Hp(+)	39	19	20	16	23	40.62 ± 10.76
Hp(−)	29	21	8	6	23	38.79 ± 10.10
SQD	28	15	13	2	26	41.75 ± 9.24
Hp(+)	13	1	12	1	12	41.84 ± 8.60
Hp(−)	15	2	13	1	14	41.66 ± 10.06
CON	14	8	6	—	—	38.75 ± 8.64

*Notes*. There is no statistical difference for the gender and age among the groups of DHSS, SQD, and CON (*P* > 0.05). Hp(+): *Helicobacter pylori* (Hp) positive; Hp(−): Hp negative; SQD: spleen-qi deficiency syndrome; CON: control group; M: male; FEM; female; PU: peptic ulcer; CG: chronic gastritis; —: none.
